# Impact of pressure on the structural, Raman, superconducting, and normal state resistivity properties of Y_5_Rh_6_Sn_18_ quasi-skutterudite single crystal

**DOI:** 10.1038/s41598-026-40887-8

**Published:** 2026-03-10

**Authors:** Govindaraj Lingannan, Muthukumaran Sundaramoorthy, Thiagarajan Maran, Anwesha Chakraborty, Chia Nung Kuo, Chin Shan Lue, Debjani Karmakar, Anna Delin, Olle Eriksson, Mahmoud Abdel-Hafiez, Sonachalam Arumugam, Boby Joseph

**Affiliations:** 1 College of General Education, University of Doha for Science and Technology, Doha, Qatar; 2 Centre for High Pressure Research, School of Physics, Bharathidasan University, Tiruchirappalli, 620024 India; 3 Elettra-Sincrotrone Trieste S.C. p. A, S.S. 14, Km 163.5 in Area Science Park, 34149 Basovizza, Italy; 4 Homi Bhabha National Institute, Anushaktinagar, Mumbai, 400085 India; 5 Department of Physics, National Cheng Kung University, Tainan, 70101 Taiwan; 6 Taiwan Consortium of Emergent Crystalline Materials (TCECM), National Science and Technology Council, Taipei, 10601 Taiwan; 7 Department of Physics and Astronomy, Uppsala University, Box 516, 751 20 Uppsala, Sweden; 8 Technical Physics Division, Bhabha Atomic Research Centre, Mumbai, 400085 India; 9 Department of Applied Physics, KTH Royal Institute of Technology, 106 91 Stockholm, Sweden; 10 Wallenberg Initiative Materials Science (WISE), Royal Institute of Technology, Stockholm, Sweden; 11 Swedish e-Science Research Center (SeRC), KTH Royal Institute of Technology, 10044 Stockholm, Sweden; 12 Department of Applied Physics and Astronomy, University of Sharjah, P. O. Box 27272, Sharjah, United Arab Emirates; 13 Tamil Nadu Open University, Chennai, 600015 India

**Keywords:** Materials science, Physics

## Abstract

High-pressure (HP) electrical resistivity measurements of superconducting quasi-skutterudite Y_5_Rh_6_Sn_18_ single crystals were conducted up to 10.7 GPa using a diamond anvil pressure cell. Additionally, HP synchrotron X-ray powder diffraction (XRPD) studies were performed on Y_5_Rh_6_Sn_18_ up to 8 GPa and Sc_5_Rh_6_Sn_18_ up to 11.7 GPa, along with HP Raman spectroscopy. At ambient conditions, the Y_5_Rh_6_Sn_18_ single crystal shows an onset 3.60 K, which increases to a maximum of 3.94 K at 7.89 GPa. Beyond this pressure, further increases lead to a slight decline in T_c_. Furthermore, the bad metal nature of the Y_5_Rh_6_Sn_18_ single crystal is suppressed by external pressure, with the residual resistivity ratio (RRR) value increasing from 0.77 to 0.92. The activation energy for electric conduction (E_a_) decreases from 2.74 meV at 0.62 GPa to 0.39 meV at 10.65 GPa, indicating enhanced metallic behaviour. Additionally, four Raman vibrational modes are observed, which exhibit a smooth blue shift with increasing pressure up to 9.6 GPa, similar to that of its sister compound Sc_5_Rh_6_Sn_18_. Both Y_5_Rh_6_Sn_18_ and Sc_5_Rh_6_Sn_18_ crystallize in the tetragonal *I*4_1_/*acd* space group. With increasing external pressure up to 7 GPa, these compounds exhibit a smooth reduction in unit-cell volume without any structural phase transitions, as described by a second-order Birch-Murnaghan equation of state. Above 7 GPa, Sc_5_Rh_6_Sn_18_ also shows an upturn in both lattice parameters a and c and unit cell volume, while an upturn in only the lattice parameter c (and consequently on unit cell volume) is observed for Y_5_Rh_6_Sn_18_ at 7 GPa. The deviation in the lattice parameter at higher pressure in Y_5_Rh_6_Sn_18_ seems to correlate with the observed decline in T_c_ with pressure. First-principles calculations under pressure reveal a moderate increase in the density of states (DOS) up to ~ 10 GPa, followed by a slight decrease for higher pressures, a trend which is similar to the observed critical temperature. Furthermore, from theory it is clear that the structure remains stable up to approximately 30 GPa.

**Supplementary Information:**

The online version contains supplementary material available at 10.1038/s41598-026-40887-8.

## Introduction

In 1980, Remeika and his co-workers discovered a new family of ternary intermetallic stannide compounds, specifically RERh_x_Sn_y_^1–4^. This structure is similar to skutterudites. These compounds have garnered significant interest in materials science due to their unique properties, which vary based on the atomic number of the constituent rare earth elements. Lighter rare earth elements (atomic numbers 57 to 63) exhibit magnetic transitions, while heavier elements (atomic numbers 64 to 71) display superconducting transitions. Additionally, these compounds crystallize in three distinct phases: Phase I (primitive cubic), Phase II (a superstructure of Phase I, pseudo-tetragonal), and Phase III (face-centered cubic)^5–7^. The phase II pseudo-tetragonal 5–6-18 series compounds (RE_5_Rh_6_Sn_18_), have recently drawn attention due to its intriguing superconducting (SC) properties under chemical doping and external pressure as well as temperature-induced phase transitions. The series of RE_5_Rh_6_Sn_18_ (RE = Tm, Y, Sc, Lu) compounds has a cage-like structure and crystallize in a tetragonal structure with a space group of *I*4_1_*/acd* (No. 142)^3,8–12^. The cage structure is formed by Sn atoms encapsulating the RE atoms, which occupy two atomic sites (8b and 32 g) in the unit cell^3,6,7^. The superconducting transition temperatures (T_c_) for these compounds are 2.2 K for Tm, 3 K for Y, 4 K for Lu, and 5 K for Sc^3,6,8–12^. However, a general picture correlating the crystal structure and SC properties in these stannides remains challenging. For instance, Sc and Lu samples are single-band nodeless *s*-wave, isotropic gap BCS superconductors^8,9^, whereas Y exhibits two-band superconductivity with an anisotropic gap and point nodes^10,11^, confirming that the RE atom strongly influences the nature of the superconducting gap.

Temperature-dependent electrical resistivity measurements of RE_5_Rh_6_Sn_18_ (RE = Y, Lu, Sc; non-magnetic rare-earth elements) compounds reveal anisotropic behavior in the normal state. The resistivity of all three compounds shows minimal temperature dependence, with the Sc_5_Rh_6_Sn_18_ compound displaying particularly pronounced anisotropy^4^. A kink-like behavior is observed at 70 K for Sc and at 120 K for the Y and Lu compounds. This behavior is thought to be due to intrinsic structural disorder within the compounds^8,13–15^.

Furthermore, the T_c_ of these quasi-skutterudite compounds is influenced by local atomic disorder, vacancies, local inhomogeneity, and the application of external chemical and physical pressure. The T_c_ of the RE_3_Rh_4_Sn_13_ and RE_5_Rh_6_Sn_18_ series compounds is enhanced by atomic disorder^16–18^. For instance, doping Ca atoms into the Y site in the Y_5−x_Ca_x_Rh_6_Sn_18_ series increases the T_c_ from 3.08 K (at x = 0) to 3.10 K (at x = 0.5), accompanied by a phase transition. This inhomogeneity contributes to an increase in the total density of states (DOS) at the Fermi level and enhances T_c_. However, increasing the Ca content above x > 1.25 results in significant inhomogeneity, causing the Y_5_Rh_6_Sn_18_ compound to separate into two phases^18^.

Prior ab-initio calculations of Y_5_Rh_6_Sn_18_ with pressure indicated an increase in DOS at the Fermi level with pressure, while calculated at discrete pressures^18^. Applying external pressure to Y_2_Ca_2_Rh_6_Sn_17.3_ results in a decrease in T_c_ at a rate of dT_c_/dP = −0.09 K/GPa^18^. Moreover, in the (Y_1−x_Lu_x_)_5_Rh_6_Sn_18_ compound, replacing Y with Lu up to x = 0.3 linearly increases T_c_^11^. Recent studies on chemical substitution in the Rh and Y sites in the Y_5−x_(Rh_5.5_M_0.5_)Sn_18_ (M = Co, Ir, Ru, Rh, Pd) compound have shown superconductivity with spin fluctuations for M = Pd and Co doping^19,20^. Similar T_c_ enhancement due to increased atomic disorder is also observed in the isostructural compound Lu_5−δ_Rh_6_Sn_18_^17^.

Furthermore, prior studies indicated that the series of RE_3_M_4_Sn_13_ (R = Sr, Ca, La, Ce; M = Rh, Ir, Co) skutterudite compounds exhibit superconductivity, which is also associated with a second-order structural transition^5^. After combining hydrostatic pressure with chemical substitution, the structural transition can be suppressed to 0 K, thereby enhancing the superconducting transition temperature (T_c_) due to the suppression of lattice instability and the softening of the phonon spectrum^21–23^. Most of the 3–4-13 quasi-skutterudite compounds show a negative pressure coefficient of T_c_, with only a few exhibiting a positive pressure coefficient^21,22,24–26^. In the case of RE_5_Rh_6_Sn_18_ (RE = Sc, Lu and Y) compounds, a positive pressure coefficient of *T*_c_ is observed^27–29^. Recently, we reported the high-pressure structural, Raman, and electrical properties of the Sc_5_Rh_6_Sn_18_ compound up to 2.5 GPa, finding a T_c_ increasing rate of dT_c_/dP = 0.1 K/GPa^27^. Later studies by Xiong et al. reported the chemical and applied pressure effects on Lu_5_Rh_6_Sn_18_, finding that T_c_ continuously increases up to 11.4 GPa at a rate of 0.13 K/GPa^28^. Prior work at discrete pressure values on the Y_5_Rh_6_Sn_18_ compound reported the value of dT_c_/dP = 0.067 K/GPa, using quasi hydrostatic pressure techniques^29^. All these findings prompted a detailed experimental and theoretical investigation of the pressure effects on the Y_5_Rh_6_Sn_18_ compound, the results of which can be easily compared with its sister compounds Sc_5_Rh_6_Sn_18_ and Lu_5_Rh_6_Sn_18_. In this work, we perform a comprehensive hydrostatic high-pressure investigation of Y_5_Rh_6_Sn_18_ using synchrotron X-ray powder diffraction, Raman spectroscopy, and electrical resistivity measurements, complemented by first-principles calculations. Unlike most previous pressure studies on Y_5_Rh_6_Sn_18_, which were carried out under quasi-hydrostatic conditions, the present measurements were performed under hydrostatic pressure, enabling cleaner tuning of the lattice and electronic band structure. We observe a clear change in the pressure evolution of lattice parameters, particularly the c-axis and c/a ratio, around ~ 7.8 GPa, which directly correlates with the pressure at which Tc reaches a maximum and subsequently starts to decrease. High-pressure Raman measurements show a systematic blue shift of phonon modes without the appearance or disappearance of new peaks, confirming the absence of any structural phase transition. High-pressure resistivity measurements further reveal a pressure-induced evolution of transport behavior, supported by changes in the residual resistivity ratio and activation energy. Together, these results establish a direct correlation between lattice anisotropy, phonon evolution, and superconductivity in Y_5_Rh_6_Sn_18_.

## Results and discussions

### Ambient pressure results

**Fig. 1:**
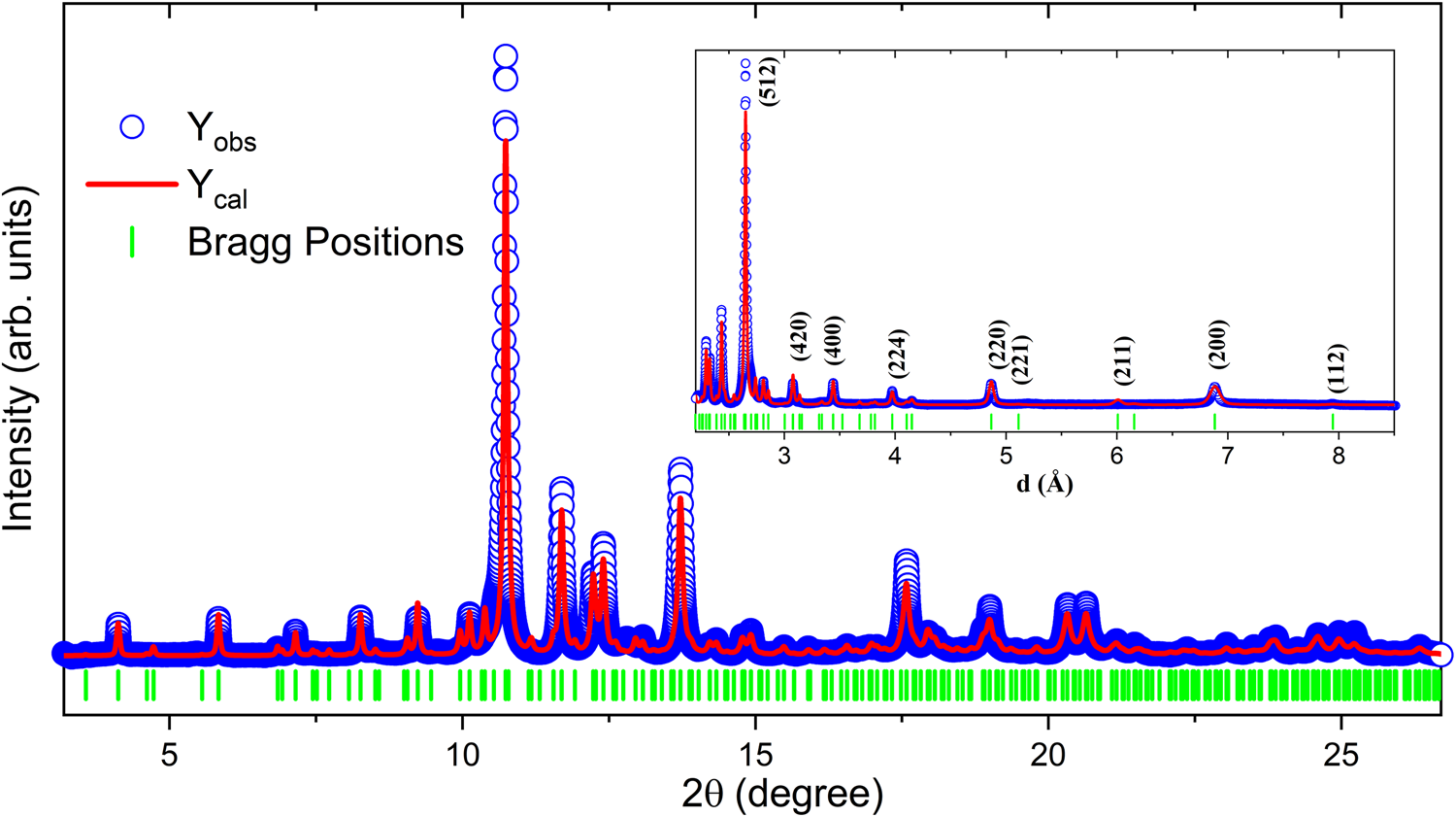
Measured powder x-ray-diffraction pattern of Y_5_Rh_6_Sn_18_ at ambient pressure and temperature together with the Rietveld refinement results. The open blue circles represent experimental data, the red solid line corresponds to the refinement fit, and the green vertical bars indicate the Bragg peak positions. The inset shows a zoomed-in view of the d-spacing range from 2.2 to 8.5 Å, highlighting the Bragg reflections.

Figure 1 shows the measured synchrotron XRPD pattern of the Y_5_Rh_6_Sn_18_ compound at ambient pressure and temperature. The results confirm that the sample has a tetragonal crystal structure with space group *I*4_1_/*acd*. The lattice parameter values are a = 13.76 Å and c = 27.5 Å. These values are in good agreement with previous reports and are higher compared with the sister compounds Lu_5_Rh_6_Sn_18_ and Sc_5_Rh_6_Sn_18_^3,7,10,27^. The differences in lattice parameters originate from the values of Y, Lu and Sc ionic radii (see Table 1). The inset figure is an enlarged view of the d-spacing pattern. The Sc_5_Rh_6_Sn_18_ ambient pressure synchrotron XRPD pattern is published in reference^27^. We did not find any impurity phases in the sample using high-resolution synchrotron XRPD data. The crystals were grown using the self-flux technique with Sn as flux; although residual Sn impurities are often present in flux-grown samples, no Sn impurity peaks were detected here, confirming the high quality of our samples. While single crystals were used for resistivity and Raman measurements, for diffraction experiments the crystals were crushed into fine powder and measured using synchrotron XRD. All diffraction data reported in this work therefore correspond to powdered samples.

**Table 1:** Comparison of R_5_Rh_6_Sn_18_ (R = Y, Lu and Sc) compounds.

	Y_5_Rh_6_Sn_18_	Lu_5_Rh_6_Sn_18_ [28]	Sc_5_Rh_6_Sn_18_ [27]
Ionic radii of RE^3+^ (pm)	90	86.1	74.5
RE atomic mass (amu)	88.91	174.97	44.96
Lattice parameter a (Å)	13.760	13.683	13.582
Lattice parameter c (Å)	27.52	27.350	27.070
Unit cell volume V (Å^3^)	5212.8	5120.6	4988.9
Bulk Modulus B_0_ (GPa)	99.79	137.43	99.27
T_c_ (K)	3.6	4.1	5.0
dT_c_/dP (K/GPa)	0.05	0.13	0.10

**Fig. 2:**
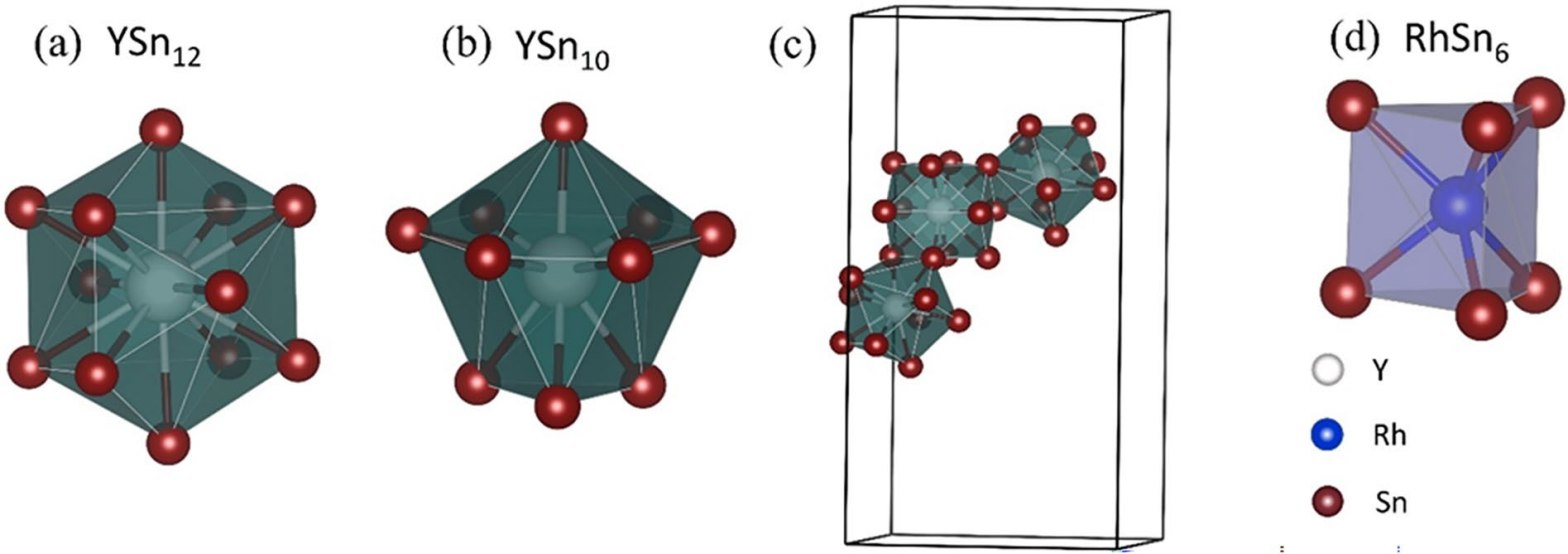
Different cage structures and their coordination in Y_5_Rh_6_Sn_18_: **a** dodecahedral Y(8b)Sn_12_ cage, **b** polyhedral Y(32 g)Sn_10_ cage, **c** edge-sharing between YSn_12_ and YSn_10_ cages, and **d** RhSn_6_ trigonal biprism cage.

Y atoms occupy the 8b and 32 g Wyckoff positions in the unit cell. The Sn atoms surrounding these sites form two types of polyhedral cages: a dodecahedral YSn_12_ cage around one Y site and a polyhedral YSn_10_ cage around the other. Additionally, Rh atoms are coordinated with six Sn atoms to form a RhSn_6_ trigonal biprism. These coordination environments are shown in Fig. 2: (1) the dodecahedral Y1(8b)Sn_12_ cage [Fig. 2(a)], which has also been reported in earlier literature^29^; (2) the Y2(32 g)Sn_10_ polyhedral cage, which shares edges with the Y1Sn_12_ cage, as illustrated in Fig. 2(b) and 2(c); and (3) the RhSn_6_ trigonal biprism, shown in Fig. 2(d), which shares faces with the YSn_12_ cage.

**Fig. 3:**
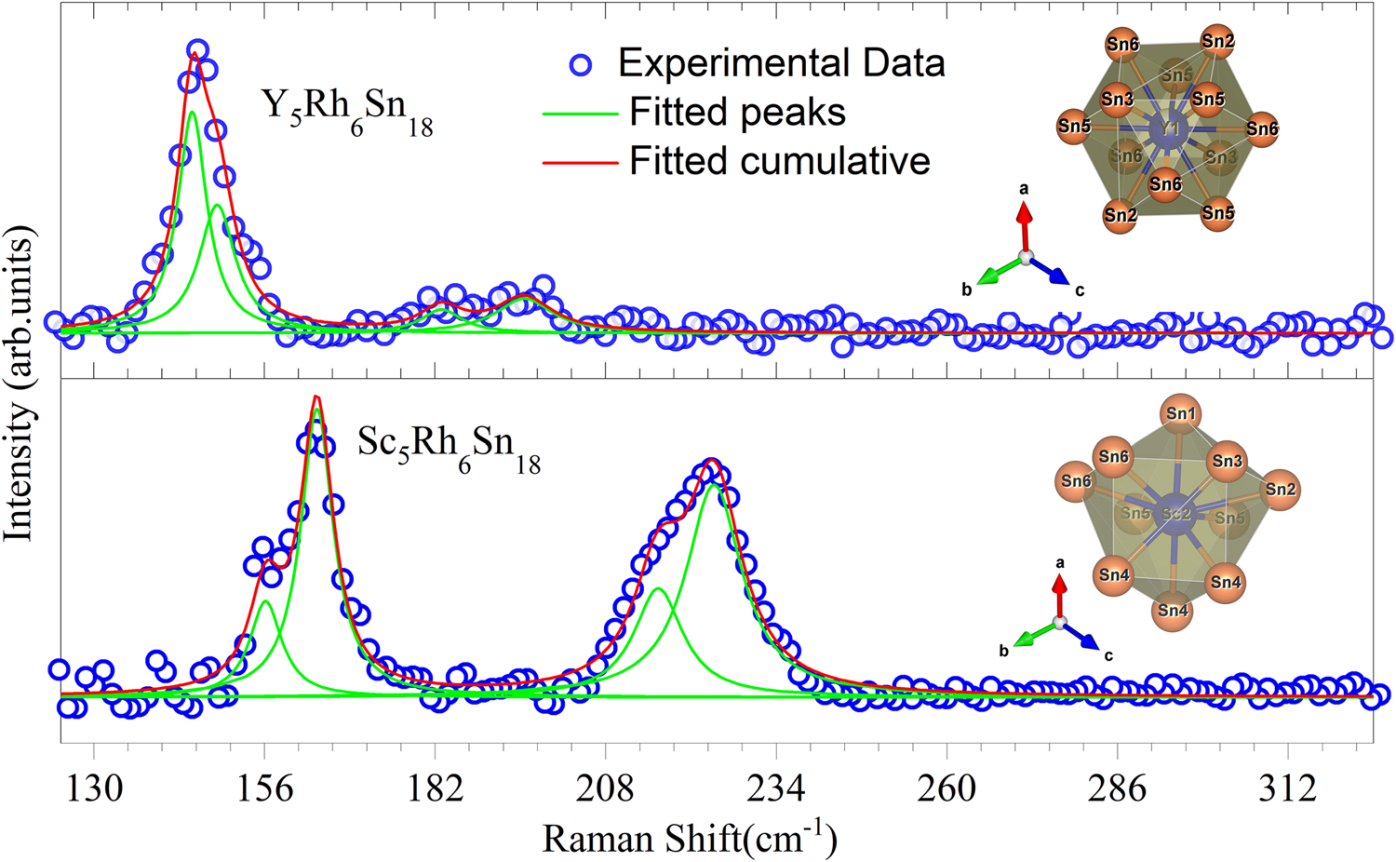
Raman spectra at ambient conditions (blue circles). The individual Lorentzian components are shown as thin green curves, while the cumulative fitted curve is shown as a thick red line. Insets show the RE-Sn polyhedra corresponding to the spectra: (top) Y1–Sn_12_ cage in Y_5_Rh_6_Sn_18_ and (bottom) Sc2–Sn_10_ cage in Sc_5_Rh_6_Sn_18_, drawn using VESTA.

Figure 3 shows the Raman spectroscopy measurements for Y_5_Rh_6_Sn_18_ and Sc_5_Rh_6_Sn_18_ single crystal at ambient conditions. Raman modes are found only in the region 125–300 cm^−1^. We found that compared to the Sc compound, the Y compound has a similar Raman signal, however, with shifts towards the lower wavenumbers. Differences in the relative intensities of the modes were also observed. This behavior can be understood as arising from two complementary factors: first, within the harmonic approximation, phonon frequencies scale inversely with the square root of atomic mass ($\:\omega\:\propto\:1/\sqrt{M}$). Since Y (88.9 amu) is nearly twice as heavy as Sc (44.9 amu), the Raman-active modes in Y_5_Rh_6_Sn_18_ are naturally shifted to lower frequencies by approximately $\:\sqrt{MSc/M}Y\approx\:0.71$. Second, due to the larger ionic radius of Y, the Y atoms expand the surrounding Sn cage relative to Sc, thereby reducing the stiffness of the lattice and further lowering the vibrational frequencies. In contrast, the smaller Sc atoms “rattle” more easily inside the Sn cage, resulting in comparatively higher Raman frequencies and intensities^30,31^.

We performed a careful spectral deconvolution using Lorentzian line-shapes to confirm the main Raman modes from the observed data. The extracted Raman mode positions and linewidths obtained from Lorentzian deconvolution for both compounds are summarized in Table 2. Raman spectroscopy studies were performed for several skutterudites compounds and reported that the Raman modes of these skutterudites are generally weak due to the metallic nature of the compounds^27,32–34^. However, though the signal were weak, we could clearly identify four Raman modes in both samples. These modes of vibrations may have originated from the RE-Sn cage structure because the RE atom produces a strong interplay between the quadrupolar moments and these quadrupolar moments could be a reason for the emergence of SC in cage compounds^34,35^. The inset figures show the Sn cages surrounding the crystallographically distinct rare-earth sites: the Y1-Sn_12_ cage in Y_5_Rh_6_Sn_18_ and the Sc2-Sn_10_ cage in Sc_5_Rh_6_Sn_18_. Previous structural studies on the RE_5_Rh_6_Sn_18_ family^14,27^ have reported that the RE1 site can accommodate partial vacancy (~ 0.92), while the RE2 site is generally fully occupied. Since both Y_5_Rh_6_Sn_18_ and Sc_5_Rh_6_Sn_18_ exhibit a similar degree of partial occupancy at the RE1 site, this factor alone cannot explain their different T_c_ values. Instead, the systematic downshift of Raman modes in Y_5_Rh_6_Sn_18_ is attributed to its heavier atomic mass and larger ionic radius, while the lighter Sc atoms enhance vibrational frequencies and may strengthen quadrupolar fluctuations and electron-phonon interactions.

**Table 2:** Raman mode positions and widths for Y_5_Rh_6_Sn_18_ and Sc_5_Rh_6_Sn_18_ obtained from the spectral deconvolution.

Raman Modes	Y_5_Rh_6_Sn_18_ (cm^−1^)		Sc_5_Rh_6_Sn_18_ (cm^−1^)	
	Position	Width	Position	Width
#1	145.0	5.4	156.2	5.9
#2	148.8	6.7	164.0	6.1
#3	183.0	7.7	216.0	9.1
#4	195.6	10.6	224.6	10.4

**Fig. 4:**
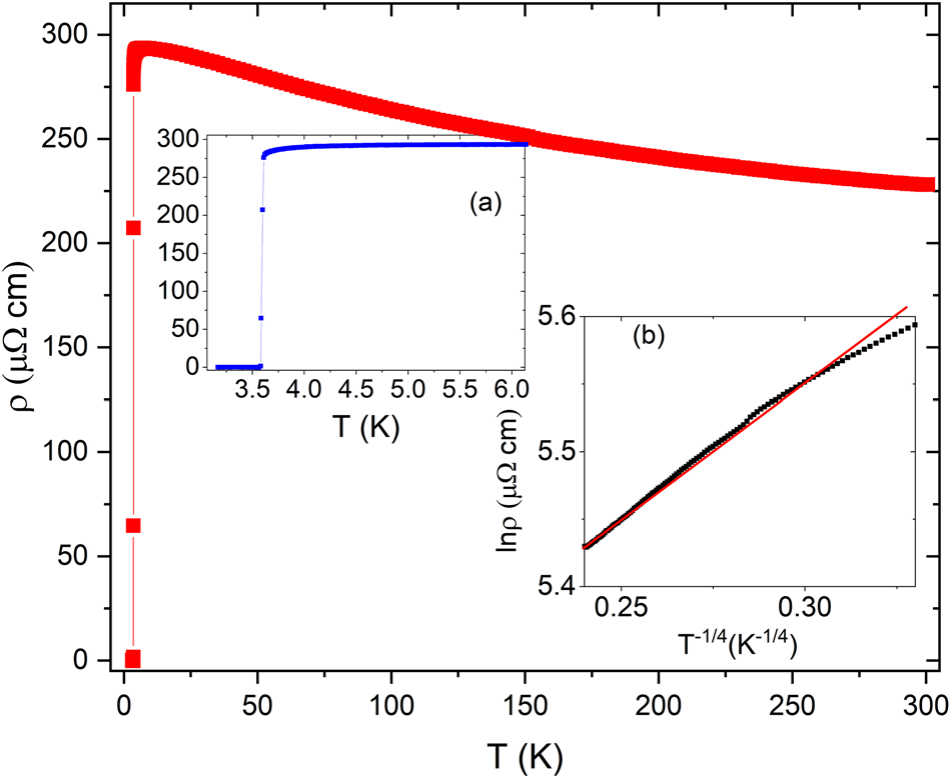
Temperature dependence of resistivity at ambient pressure from 3.2 to 300 K of single crystalline Y_5_Rh_6_Sn_18_. Inset **a** is a zoom over the 3.2 to 6 K showing a clear superconducting transition and **b** ln ρ vs. T^− 1/4^ plot for Y_5_Rh_6_Sn_18_ from 120 K to 300 K, demonstrating characteristics ofMott variable-range hopping conduction (straight line – model, symbols – experiment).

Figure 4 shows the electrical resistivity of single crystalline Y_5_Rh_6_Sn_18_ as a function of temperature under ambient pressure conditions. The material exhibits a bad metal nature, characterized by a negative temperature coefficient of resistance^13,15^. At 300 K, the resistivity is 228.08 µΩ cm. As the temperature decreases, the resistivity gradually increases, reaching a maximum value of 293.65 µΩ cm at 6 K, with a RRR value of 0.77, which confirms the bad metal behaviour. Upon further cooling, the resistivity starts to decrease, with an onset superconducting transition temperature (T_c_) at 3.60 K. The T_c_ is determined from the intersection of two extrapolated lines, T_c_^mid^ is defined as the point where the resistivity has dropped by 50%, and T_c_^off^ is where the resistivity reaches zero, for the present case, the T_c_^mid^ is 3.585 K, and T_c_^off^ is at 3.58 K. Within the temperature range of 120–300 K, the resistivity follows Mott’s three-dimensional variable-range hopping (VRH) model, ρ ~ exp[(∆M/k_B_T)^1/4^], which is characteristic of disordered materials^17,28^, as shown in the inset of Fig. 1(b). This temperature window was chosen because it is the interval where lnρ vs. T^− 1/4^ shows the clearest linearity; outside this range the plot deviates from linear behavior. The superconducting width (ΔT_c_ = 0.02 K) of the Y_5_Rh_6_Sn_18_ is found to be very sharp. The T_c_ at ambient pressure is consistent with previous reports and is notably higher than the values reported^10,11,13,17^. The sister compound, Sc_5_Rh_6_Sn_18_ exhibits metallic resistivity behavior down to the superconducting transition, while Lu_5_Rh_6_Sn_18_ displays properties intermediate between Sc_5_Rh_6_Sn_18_ and Y_5_Rh_6_Sn_18_. Among these compounds, the Y atom has the largest ionic radius, which results in an expanded unit cell volume and influences the electronic bandwidth and DOS at the Fermi level. Conversely, Sc has the smallest ionic radius, corresponding to a more compressed unit cell and a more metallic-like conductivity. Lu, with its intermediate ionic radius and cell volume, shows transport properties that lie between those of the Sc- and Y-based compound. We emphasize that while ionic size provides a useful heuristic to rationalize differences in unit cell volume, a full microscopic understanding requires electronic structure calculations. Indeed, our first-principles results confirm that the observed transport behavior correlates with the pressure- and volume-dependent evolution of the DOS. A comparative resistivity plot for Y_5_Rh_6_Sn_18_ at ambient and 10.6 GPa pressure and Sc_5_Rh_6_Sn_18_ compound is provided in the Supplementary Information (Figure S1) for better understanding. All members of this family show only weak temperature dependence of resistivity, consistent with intrinsic structural disorder^8,13,15,27^.

### High pressure synchrotron powder X-ray diffraction

**Fig. 5:**
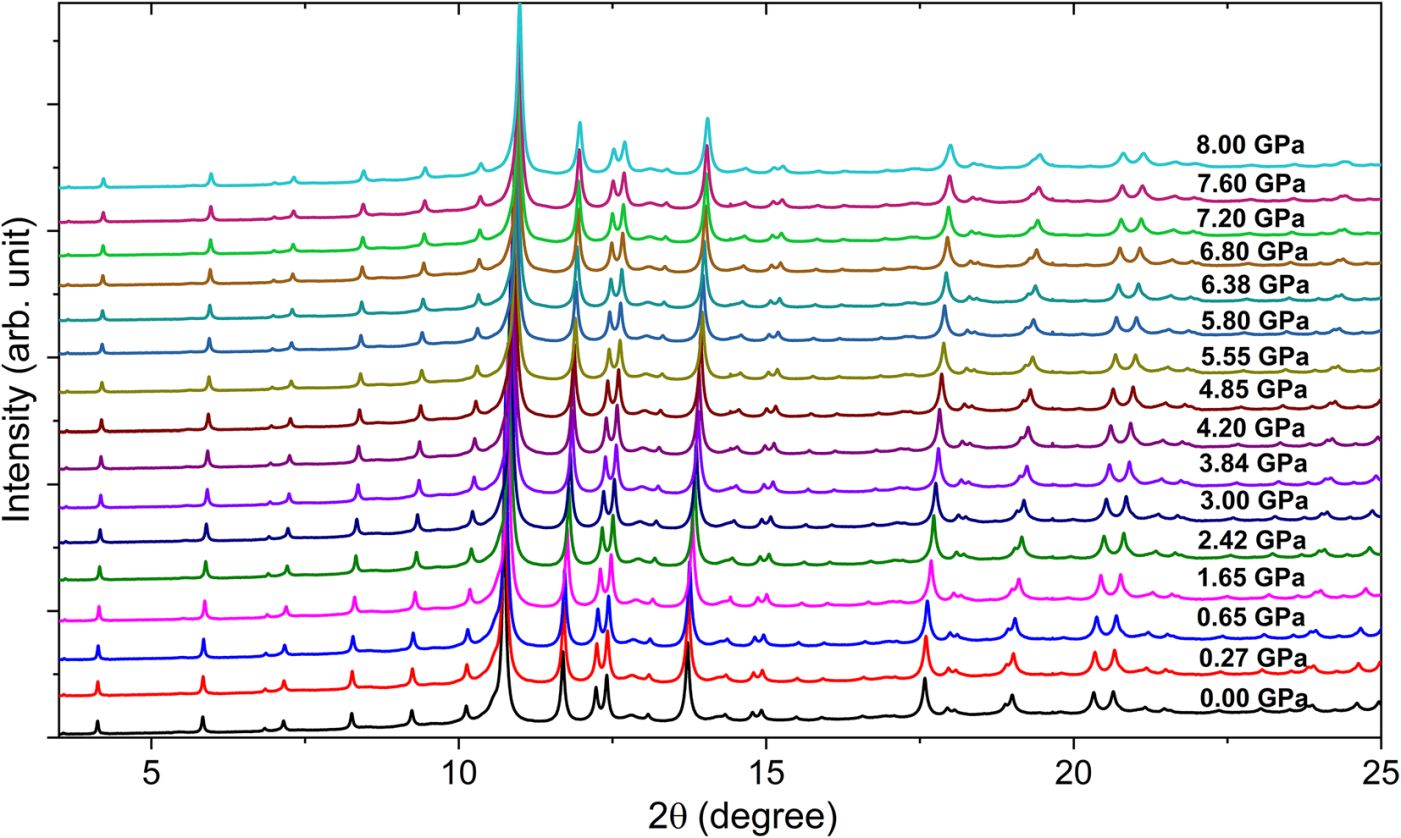
Measured HP-XRPD patterns of Y_5_Rh_6_Sn_18_ up to 8 GPa. 0.00 GPa stands for ambient pressure

The synchrotron HP-XRPD patterns of Y_5_Rh_6_Sn_18_, measured at several pressures up to 8 GPa at room temperature, are presented in Fig. 5. The corresponding patterns for Sc_5_Rh_6_Sn_18_ up to 11.7 GPa are available in supplementary information (Figure S2). As the pressure increases, the diffraction peaks shift towards higher angles due to the linear decrease in d-spacing, while maintaining the tetragonal phase across the measured pressure range (0 to 8 GPa for Y_5_Rh_6_Sn_18_ and 0 to 11.1 GPa for Sc_5_Rh_6_Sn18). The high-resolution synchrotron HP-XRPD data further substantiates this stability. No new peaks emerged, nor did any existing peaks disappear, indicating no symmetric changes or anomalies within this pressure range. This confirms that both compounds preserve their tetragonal crystal structure within the said pressure range.

**Fig. 6:**
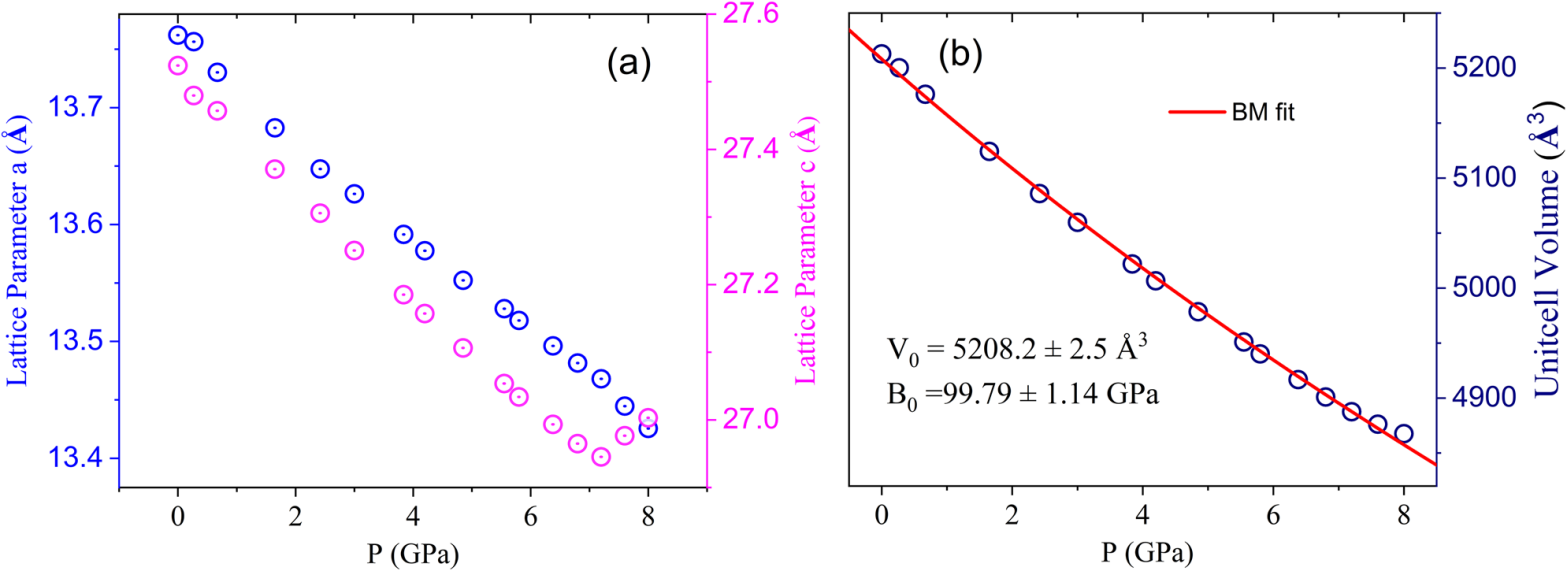
**a** Represents the Y_5_Rh_6_Sn_18_ lattice parameters as a function of pressure. **b** Depicts unit cell volume as a function of pressure. The red solid line represents the second-order Birch-Murnaghan equation of state fitting.

The pressure dependence of the lattice parameters and unit cell volume of the Y_5_Rh_6_Sn_18_ compound is illustrated in Fig. 6(a) and (b). The red solid lines represent the fitting results from the Birch-Murnaghan equation. This fitting yielded V_0_ = 5208.2 Å^3^ and a bulk modulus B_0_ = 99.79 GPa for Y_5_Rh_6_Sn_18_, and V_0_ = 4975.9 Å^3^ and B_0_ = 114.96 GPa for Sc_5_Rh_6_Sn_18_. The lattice parameters and unit cell volume of the Sc_5_Rh_6_Sn_18_ compound are available in the Supplementary Information (Figure S3). For Sc_5_Rh_6_Sn_18_, the c/a ratio remains nearly constant up to ~ 7 GPa (~ 1.99) and then begins to decrease (Figure S4). In the case of Y_5_Rh_6_Sn_18_, the c/a ratio is flat up to ~ 7.2 GPa (~ 2), after which it shows a clear upward deviation (Figure S5). These anomalies coincide with the pressure regime where T_c_ begins to decrease, confirming the strong correlation between lattice anisotropy and superconductivity. This observation suggests that the suppression of T_c_ at higher pressures is linked to an anisotropic lattice response, particularly along the c-axis, rather than to a structural phase transition. Such anisotropic compression is expected to modify the cage geometry and lattice dynamics that are relevant for superconducting pairing in Y_5_Rh_6_Sn_18_.

Substituting Sc atoms for Y in the RE_5_Rh_6_Sn_18_ compound results in a volume decrease due to the chemical pressure effect and a slight increase in the bulk modulus. The corresponding lattice parameters, unit cell volumes, and bulk moduli are summarized in Table 1. Notably, the lattice parameter c shows a sudden rise in the Y_5_Rh_6_Sn_18_ sample, which is reflected in the unit cell volume as a function of pressure, coinciding with changes in T_c_ observed in resistivity measurements. Similarly, the Sc_5_Rh_6_Sn_18_ compound exhibited anomalies in both lattice parameters a and c, which are reflected in the pressure-dependent unit cell volume. This behaviour is also observed in the Lu_5_Rh_6_Sn_18_ compound, although it was not carefully noted by the authors^28^. This trend in the unit cell volume as a function of pressure affects the T_c_ enhancement, which starts to decline above 7.89 GPa.

Furthermore, the Lu_5_Rh_6_Sn_18_ compound showed an increase in T_c_ up to 12 GPa under quasi-hydrostatic pressure. In our Y_5_Rh_6_Sn_18_ resistivity data, which were obtained under hydrostatic conditions, the pressure effectively impacted the sample, leading to a decreasing trend in T_c_ above 7.89 GPa.

### High pressure Raman spectroscopy

**Fig. 7:**
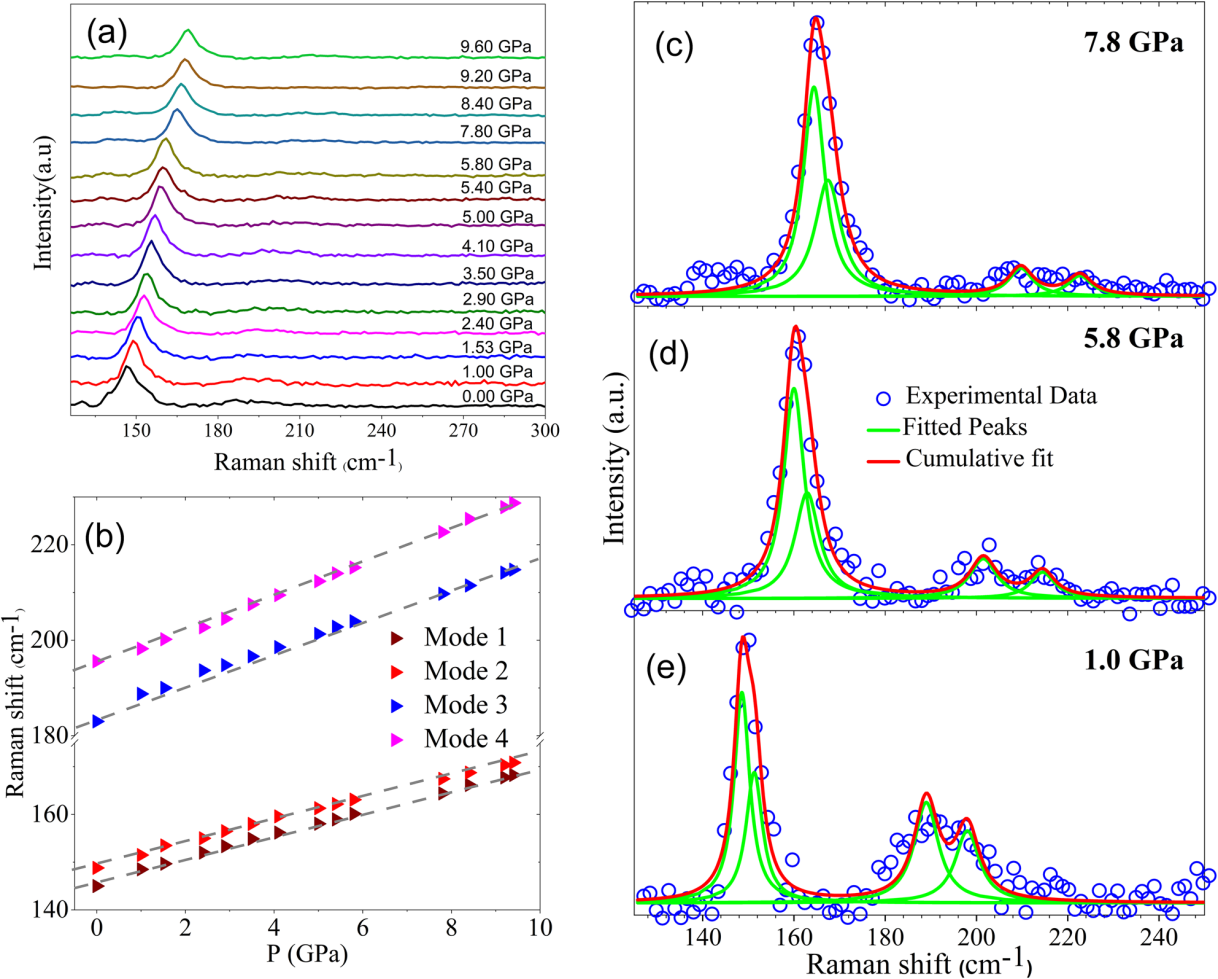
**a** Measured Raman spectrum of Y_5_Rh_6_Sn_18_ single crystal under various pressures up to 9.6 GPa, with measured curves shifted vertically for clarity. **b** Raman modes #1 (145 cm^−1^) (wine triangle), #2 (148.8 cm^−1^) (red triangle),#3 (blue triangle) (183.0 cm^−1^) and #4 (195.6 cm^−1^) (pink triangle) as a function of pressure up to 9.6 GPa. The grey dashed lines are a linear fit. **c**–**e** Raman Spectrum of Y_5_Rh_6_Sn_18_ at 7.8 GPa, 5.8 GPa and 1 GPa with the result of spectral deconvolution.

The HP Raman spectra of Y_5_Rh_6_Sn_18_ are shown in Fig. 7. Figure 7(a) presents a stacked plot of the measured HP Raman spectra up to 9.6 GPa, while Fig. 7(b) illustrates the Raman mode of vibration as a function of pressure. As the pressure increases, the Raman modes shift towards higher wavenumbers, and no anomalies are observed. The pressure dependence of Raman modes #1 (145 cm^−1^) (wine triangle), #2 (148.8 cm^−1^) (red triangle),#3 (blue triangle) (183.0 cm^−1^) and #4 (195.6 cm^−1^) (pink triangle) is presented in Fig. 7(b). Within the resolution of our measurements, these Γ-point modes exhibit a linear increase with pressure. The grey dashed lines represent the linear fit to the data. This finding rules out any possible structural phase transition and robustness in this compound. Therefore, the pressure-induced changes in superconductivity must arise from continuous lattice evolution and anisotropic stiffening effects, rather than from symmetry breaking or structural reconstruction.

### High pressure electrical resistivity

**Fig. 8:**
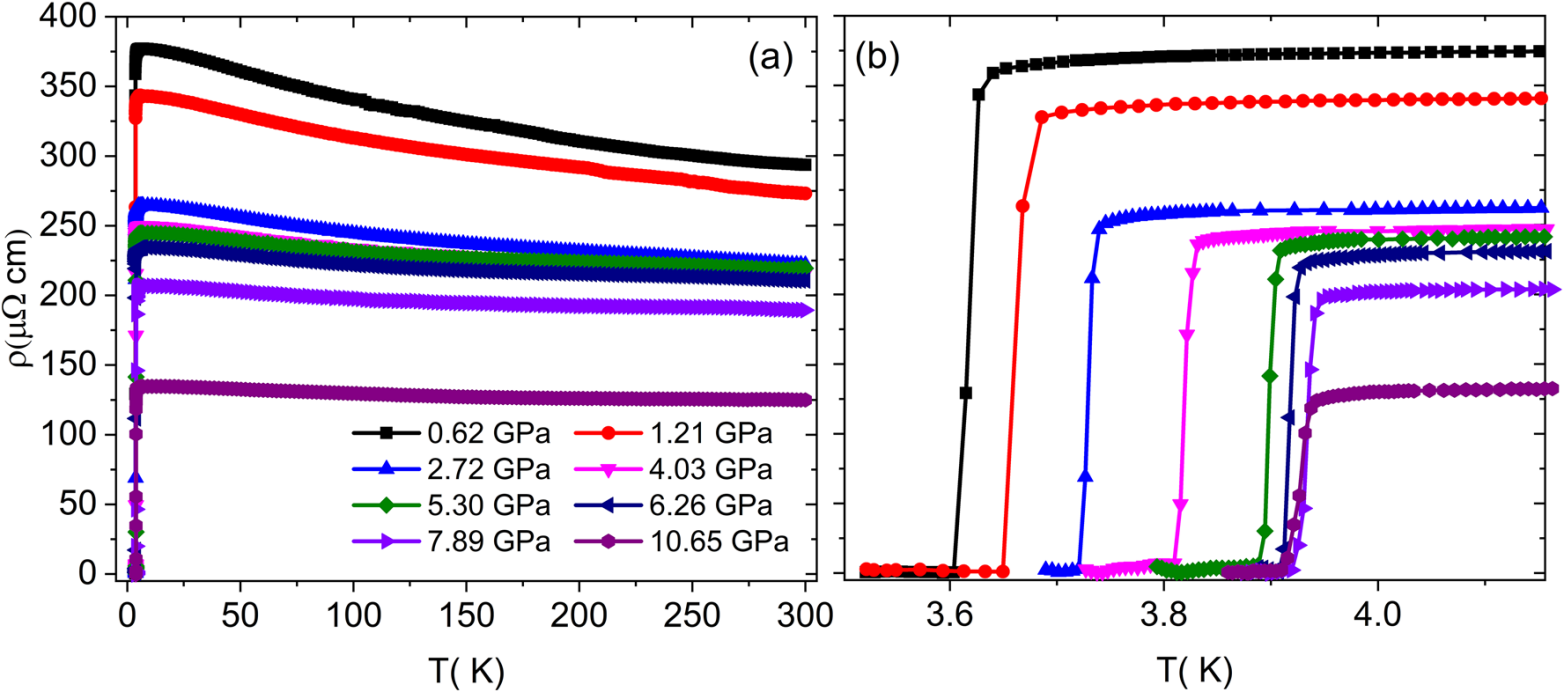
**a** Temperature-dependence of ρ at various fixed pressures. **b** Enlarged view of the superconducting transition region.

Figure 8(a) shows ρ as a function of temperature under various applied pressures. At 0.62 GPa, the Y_5_Rh_6_Sn_18_ compound exhibits bad metal, or an incoherent metal behavior followed by a superconducting transition^13,15^. As the pressure increases, the resistivity decreases monotonically, and the bad metal behavior is almost suppressed at 10.65 GPa (RRR = 0.92) due to the increase in the DOS at the Fermi level [N(E_F_)], similar pressure enhanced T_c_ and DOS is observed in Ba_6_Ge_25_ and Ba_4_Na_2_Ge_25_ cage compounds^36^. Figure 8(b) provides an enlarged view of the superconducting region. The T_c_ is enhanced with the application of pressure up to 7.89 GPa, beyond this pressure, T_c_ starts to decrease.

**Fig. 9:**
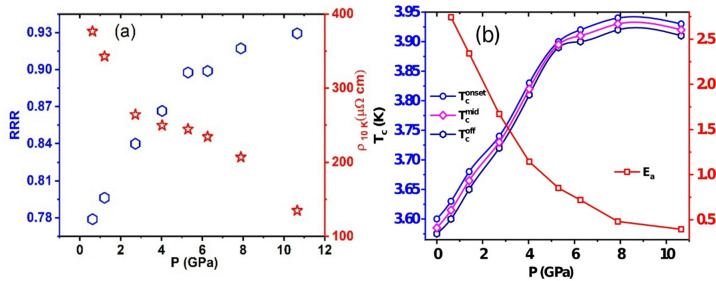
**a** Pressure dependence of RRR value and ρ at 10 K; **b** The superconducting transition temperatures (T_c_^on^, T_c_^mid^ and T_c_^off^) and activation energy (E_a_) as a function of pressure.

Figure 9(a): displays the changes in the RRR value and ρ at 10 K under varying pressures. As pressure rises, the RRR value increases from 0.77 to 0.92, while ρ at 10 K decreases from 376.48 µΩ cm to 134.74 µΩ cm. Between 2 and 6 GPa, the decline in ρ at 10 K slows down. These observations suggest a rise in the DOS at the Fermi level, which enhances electrical conduction. Overall, there is a clear trend of decreasing electrical resistivity with increasing pressure, indicating reduced electron-phonon scattering and improved conduction.

Figure 9(b) illustrates the pressure dependence of T_c_^on^, T_c_^mid^, and T_c_^off^ and activation energy (E_a_). At ambient pressure (labelled as 0 or 0.00 GPa), 0 GPa, T_c_^on^, T_c_^mid^, and T_c_^off^ are 3.60 K, 3.58 K, and 3.59 K, respectively. With increasing pressure, T_c_ shows a positive temperature coefficient, shifting to higher temperatures up to 7.89 GPa. The T_c_ increasing rate is dT_c_/dP = + 0.05 K/GPa, which is half the rate observed in its sister compounds, Sc_5_Rh6Sn_18_ and Lu_5_Rh_6_Sn_18_, with dT_c_/dP values of + 0.1 K/GPa and + 0.13 K/GPa, respectively. The T_c_ increment rate of Lu_5_Rh_6_Sn_18_ is expected to be intermediate between Y_5_Rh_6_Sn_18_ and Sc_5_Rh_6_Sn_18_. The HP electrical studies on Lu_5_Rh_6_Sn_18_ were conducted under quasi-hydrostatic pressure conditions using cubic boron nitride (BN) as a pressure-transmitting medium (PTM)^28^. This methodological difference could explain the variations, as the current research on Y_5_Rh_6_Sn_18_ and Sc_5_Rh_6_Sn_18_ was carried out under hydrostatic pressure conditions^27^.

We also calculated the thermal activation energy of electrical conductivity (E_a_) as a function of pressure, which is consistent with the RRR and ρ at 10 K. Using the thermal activation formula

$$\:{\uprho\:}\left(T\right)={{\uprho\:}}_{0}\mathrm{exp}\left(\frac{{E}_{a}}{{k}_{B}T}\right)$$

where E_a_ is the thermal activation energy gap, ρ_0_ is a prefactor, and k_B_ is Boltzmann’s constant^37–39^. We fitted the ρ(T) data in the temperature range of 150–300 K. The E_a_ value decreased from 2.74 meV at 0.62 GPa to 0.39 meV at 10.65 GPa. The pressure-dependent activation energy values extracted from this fitting are summarized in Fig. 9(b). This reduction in activation energy indicates enhanced metallic behaviour, supported by E_a_ and RRR studies under various pressures^40,41^. The pressure changes the RE-Sn cage size simultaneously enhances the carrier concentration in RE_5_Sn_6_Sn_18_ compounds.

Under ambient pressure conditions, the Y_5_Rh_6_Sn_18_ compound, containing the rare-earth (RE) ion with the largest ionic radius, exhibits the highest unit cell volume and the lowest superconducting transition temperature (T_c_). In contrast, the Lu_5_Rh_6_Sn_18_ compound, with an intermediate ionic radius, shows intermediate values of unit cell volume, T_c_, and electrical conductivity. The compound with the smallest RE ionic radius (Sc) displays the lowest unit cell volume, highest T_c_, and most metallic behaviour. This trend is attributed to the enhanced ‘rattling’ of the RE atom within the structural cage, which becomes more pronounced as the unit cell volume decreases. Such compression increases the density of states (DOS) at the Fermi level. A similar evolution is observed under applied pressure across all RE_5_Rh_6_Sn_18_ compounds, where both the RRR and the E_a_ confirm that DOS increases with pressure. The rate of DOS enhancement, however, tends to saturate above ~ 7 GPa, coinciding with the pressure range where T_c_ reaches its maximum. At higher pressures, superconductivity is gradually suppressed, which we attribute to anisotropic lattice stiffening and the associated weakening of anharmonic rattling modes, rather than to a further reduction in the electronic density of states^27–29^. Once the DOS enhancement saturates, further pressure-induced anisotropic lattice changes, particularly along the c-axis, can dominate the superconducting response, leading to the observed suppression of Tc at higher pressures.

These observations align with the general trend that increased pressure results in a higher density of states at the Fermi level, thus enhancing superconductivity. Therefore, our findings indicate that the T_c_ enhancement under pressure is closely related to the changes in the electronic structure and density of charge carriers in Y_5_Rh_6_Sn_18_.

### First-principles investigation under high pressure

**Fig. 10:**
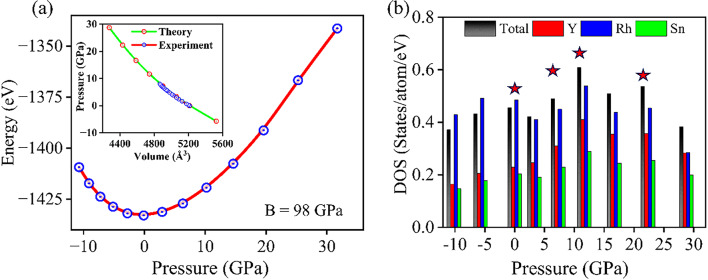
**a** Variation of ground state total energy under pressure with pressure-volume isotherm as inset, **b** comparison of total and partial DOS at Fermi energy by using tetrahedron method, ★ denotes the extrema of DOS.

We have studied the compression and the expansion of the unit cell starting from the experimental value of the equilibrium volume as 5212.8 Å^3^ at ambient pressure and computed the total ground-state energy. The total energy versus volume plot is used to determine the pressures corresponding to both compression and expansion after fitting it with the Birch Murnaghan equation of states representing the pressure-volume relation as^42^:

$$\:P\left(V\right)\:=\:\frac{3{B}_{0}}{2}\left[\:\right({\frac{{V}_{0}}{V})}^{\frac{7}{3}}\:-\:\left({\frac{{V}_{0}}{V})}^{\frac{5}{3}}\:\right]\{1+\:\frac{3}{4}\left({B}_{0}^{{\prime\:}}-\:4\right)\left[\left({\frac{{V}_{0}}{V})}^{\frac{2}{3}}-1\right]\right\},$$

Where *P* is the pressure, *V*_*0*_ is the equilibrium volume at ambient pressure, *V* is the changed volume, *B*_*0*_ signifies the bulk modulus, and $\:{B}_{0}^{{\prime\:}}$ is the derivative of the bulk modulus at ambient pressure. The ground state energy of the relaxed structures increases from a minimum value for both compression and expansion, as shown in Fig. 10(a) The theoretically fitted pressure versus volume curve shows a close trend as the experimental one, as displayed at the inset of Fig. 10(a). The Bulk modulus found from the theoretical fitting is 98 GPa, which resembles well with the value obtained from the experimental curve as 99.79 GPa.

In Fig. 10(b) the total and partial DOS for 4*d* states of Y and Rh and 5*p* states of Sn at Fermi energy level (*E*_*F*_) are compared under pressure by using the tetrahedron method. Both the total and partial DOS at *E*_*F*_ undergo an initial moderate decrease up to 3.3 GPa followed by an increase up to 11.7 GPa and a subsequent decrease beyond 11.7 GPa. We have selected (marked with * in Fig. 10(a) and (b)) four different pressures where the DOS at *E*_*F*_ display an extremum value. Next, we investigate the total and partial DOS by using Gaussian smearing method and the corresponding band structures under these four marked pressures.

**Fig. 11:**
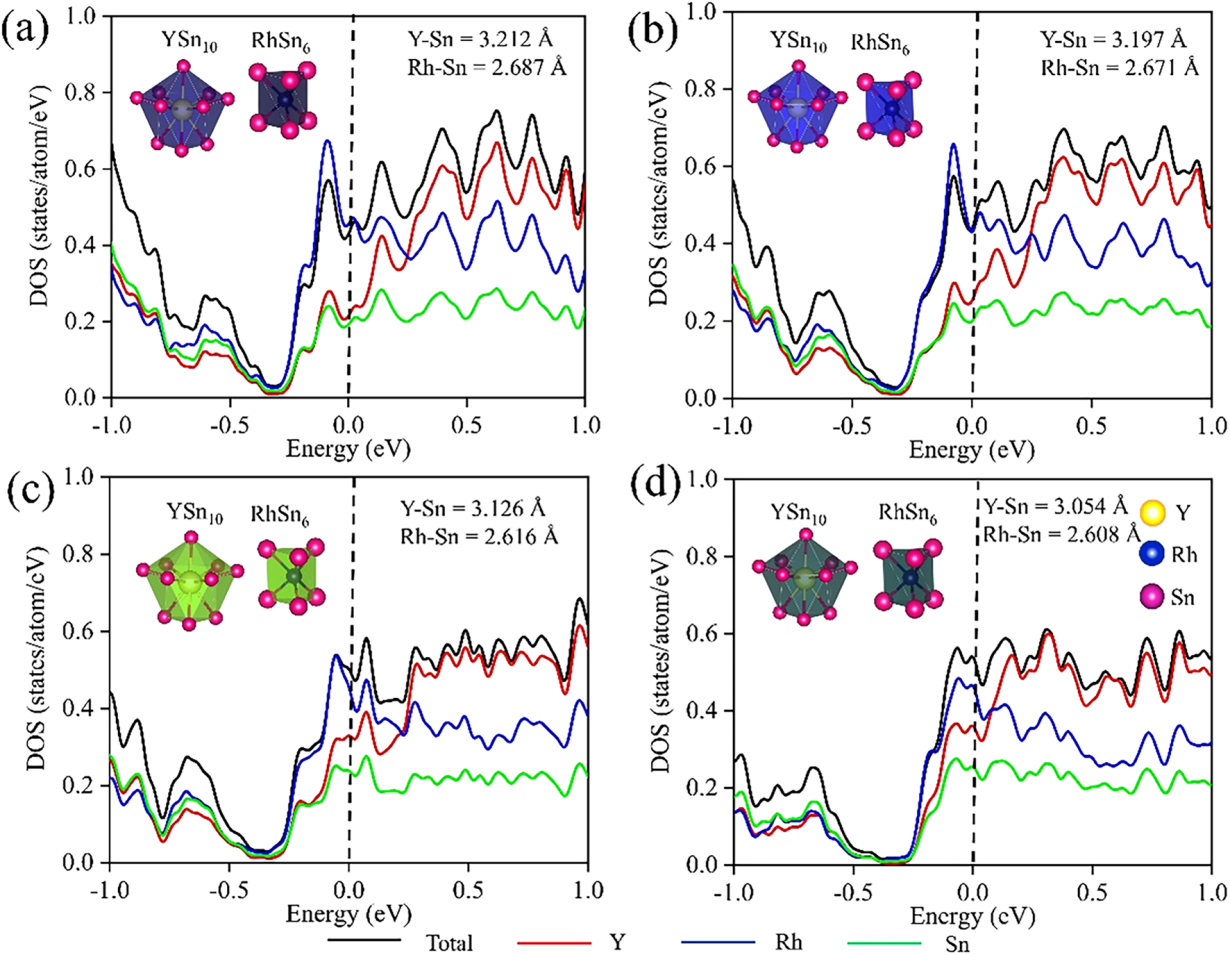
Density of states per atom at pressure **a** 0 GPa, **b** 7.3 GPa, **c** 11.7 GPa, **d** 22.3 GPa. The spectra have been broadened using Gaussian smearing.

The evolution of the DOS per atom with increasing pressure reveals a significant contribution from Rh-4*d* states at the *E*_*F*_, as shown in Fig. 11(a)–(d). With an initial moderate increase in pressure, the shallow pseudogap-like feature near *E*_*F*_ observed at ambient pressure becomes more pronounced in both the total and partial DOS, indicating an enhanced electronic localization. With further increase of pressure, both the total and partial DOS display a small peak near *E*_*F*_. A close inspection near *E*_*F*_ reveals the more prominent impact of Y and Rh-4*d* states than the Sn-5*p* states.

**Fig. 12:**
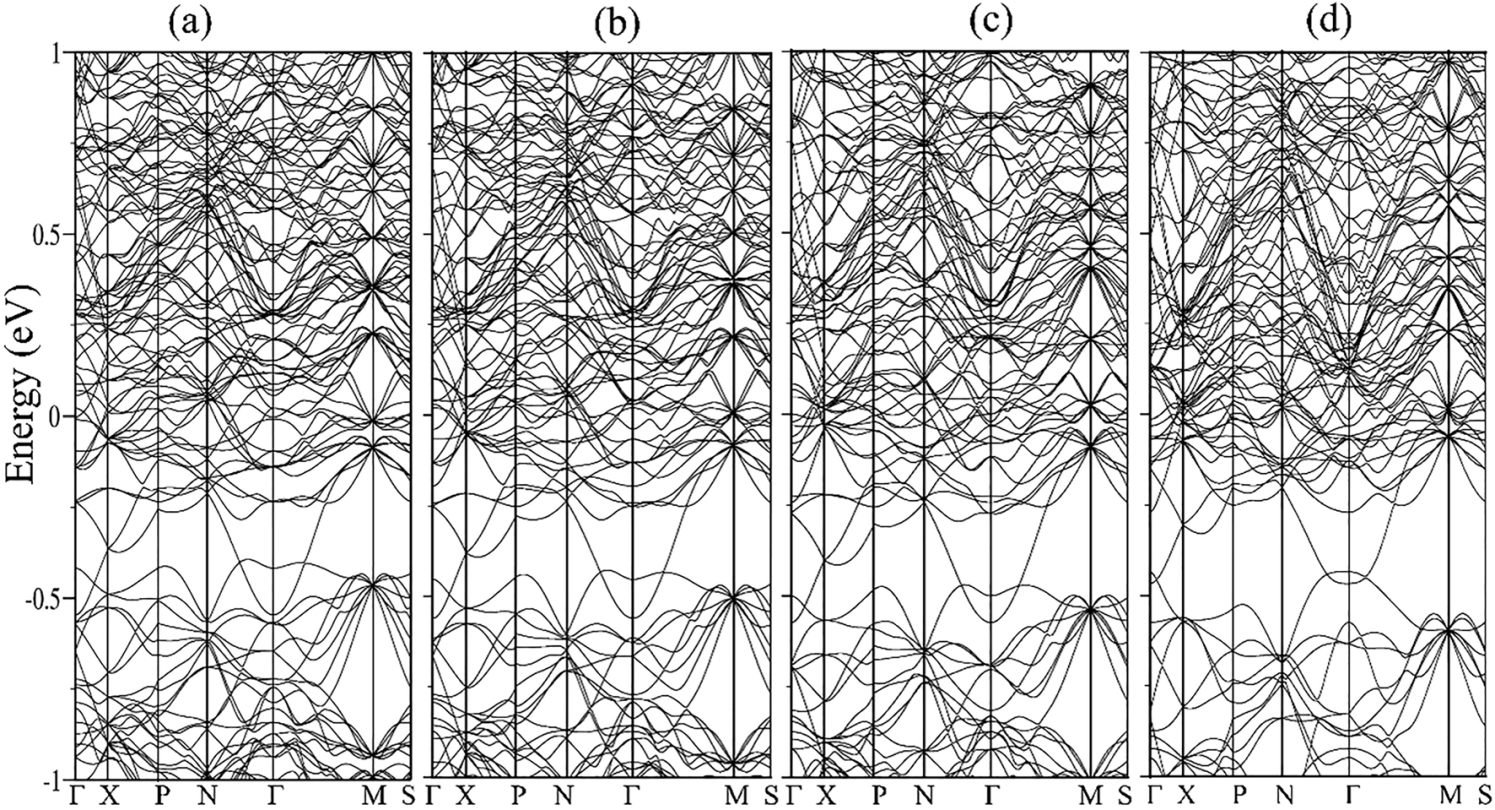
Band structures of Y_5_Rh_6_Sn_18_ at pressures **a** 0 GPa, **b** 7.3 GPa, **c** 11.7 GPa, **d** 22.3 GPa.

A survey of the structural impacts under high pressure reveals that the Y-Sn bond lengths are more compressible compared to the Rh-Sn bonds. The decrease in bond length is observed from 3.212 Å to 3.054 Å for Y-Rh and from 2.687 Å to 2.608 Å for Rh-Sn after application of a pressure of 22.3 GPa starting from the ambient pressure. The bond-lengths corresponding to the cage polyhedra under different conditions of pressure are mentioned in Fig. 11 (a)-(d). The corresponding band structures of Y_5_Rh_6_Sn_18_ are presented in Fig. 12 (a)-(d), where, with a gradual increase of pressure, a simultaneous increase of the density of bands near *E*_*F*_ is observed. Therefore, the electronic structure of Y_5_Rh_6_Sn_18_ indicates a prominent impact with a variation of the applied pressure, which, in due course, will impact its superconducting properties.

**Fig. 13:**
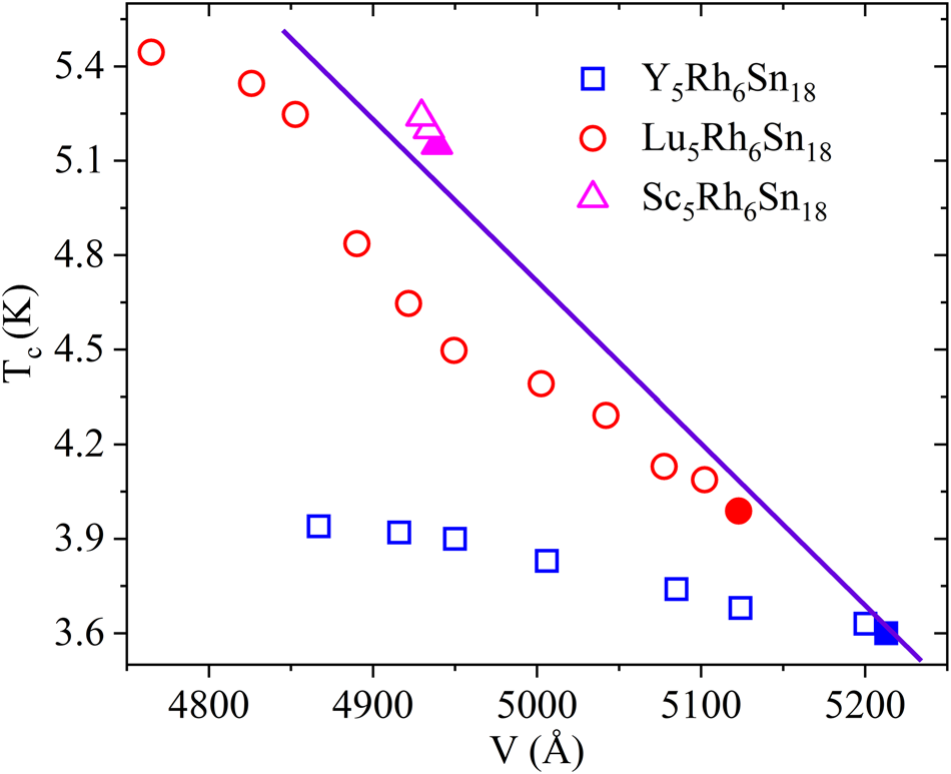
Variation of the T_c_ as a function of V for R_5_Rh_6_Sn_18_ (R = Y, Lu, Sc). Blue squares represent Y_5_Rh_6_Sn_18_ (this work), red circles represent Lu_5_Rh_6_Sn_18_ (data extracted from Ref^28^, and pink rectangles represent Sc_5_Rh_6_Sn_18_ (Ref^27^. Filled symbols correspond to ambient pressure and open symbols to high pressure. For Y_5_Rh_6_Sn_18_, T_c_(P) is obtained from resistivity measurements, while the corresponding unit-cell volume V(P) is calculated using the EOS fit to the XRPD data, evaluated at the resistivity pressure points. The straight line shows the ambient-pressure trend of Tc with unit-cell volume.

Based on the results of high-pressure ρ(T) and structural investigations, we constructed the superconducting critical temperature (T_c_) - unit cell volume phase diagram for RE_5_Rh_6_Sn_18_ (RE = Y, Lu, Sc) compounds, as shown in Fig. 13. Replacing the RE atom from a higher ionic radius element (Y) to a lower ionic radius element (Sc) result in a linear decrease in unit cell volume due to the chemical pressure effect. Concurrently, T_c_ increases with decreasing unit cell volume under chemical pressure (denoted by closed symbols). The violet linear line illustrates the linear enhancement of T_c_ due to chemical pressure. The unit cell volume and corresponding T_c_ values are tabulated in Table 1 for ambient pressure.

The superconducting properties of these compounds depend significantly on the size of the RE atom and the bond distance between the RE and Sn atoms in the cage. From high-pressure ρ(T) measurements, we found that T_c_ increases with pressure up to 2.5 GPa for Sc_5_Rh_6_Sn_18_^27^ where further high- pressure studies are need to follow well the HPtrend. However, above 7.89 GPa, T_c_ for Y_5_Rh_6_Sn_18_ starts to decrease, while for Lu_5_Rh_6_Sn_18_, T_c_ continues to increase up to 11.4 GPa^28^.

HP-XRPD measurements of Sc_5_Rh_6_Sn_18_ and Y_5_Rh_6_Sn_18_ show that the lattice parameter decreases linearly with pressure up to approximately 7 GPa, beyond which a trend change is observed in both the compounds. This change may have a bearing on T_c_. In the case of Y_5_Rh_6_Sn_18_, it seems to cause a decrease. In Fig. 13, the open symbols indicate the T_c_ obtained from HP ρ(T) measurements corresponding to HP volume. It is to be noted that atomic disorder, lattice defects, change in the density of states at the Fermi level, and phonon stiffening all have a bearing on the T_c_^16–19,28,29^. However, further studies combining experiments and theory are needed to explain the observed pressure dependence of the superconducting transition of Y_5_Rh_6_Sn_18_. Additionally, a high-pressure electrical resistivity study on Sc_5_Rh_6_Sn_18_ covering higher pressures is also desirable to understand the mechanism of T_c_ in this series of compound.

## Conclusions

This study presents ambient and high-pressure electrical resistivity results for Y_5_Rh_6_Sn_18_ and compares those with its sister compounds Lu_5_Rh_6_Sn_18_ and Sc_5_Rh_6_Sn_18_. The experimental data are followed by ab-initio theory that in general compare well with experimental observations. Among the studied compounds, Sc_5_Rh_6_Sn_18_, with the smallest atomic radii and the smallest unit cell volume, exhibited better metallic behaviour and highest superconducting T_c_. These observations confirm that in this series, the rare-earth ionic radius plays a decisive role in determining T_c_ and conductivity through its influence on unit cell volume, lattice dynamics, and the electronic density of states near the Fermi level. HP ρ(T) results on Y_5_Rh_6_Sn_18_ indicate that T_c_ increases up to a maximum of 3.94 K at 7.89 GPa, beyond which T_c_ begins to decrease. The enhancement in T_c_ is attributed to the compression of the unit cell volume, which concurrently increases the DOS at the Fermi level, similar to the effects observed under chemical pressure and is observed in the theoretical calculations. Additionally, high-pressure Raman measurements up to 9.6 GPa show a linear increase inthe frequency of the four observed Raman modes with pressure, like the behaviour seen in the isostructural compound Sc_5_Rh_6_Sn_18_. Within the studied pressure range, the accessible Raman modes exhibited a smooth linear shift with pressure, with no anomalies detected. The downturn of T_c_ beyond 7.89 GPa may be related to changes in the c-axis lattice parameter and to the saturation of DOS growth under higher pressures.

## Methods

### Experimental methods

The Y_5_Rh_6_Sn_18_ and Sc_5_Rh_6_Sn_18_ single crystals were synthesized using the conventional Sn-flux method. Detailed synthesis procedures and ambient condition physical characterizations are provided in reference^13,27^.

Ambient and HP-XRPD studies were performed using monochromatic synchrotron radiation (λ = 0.4957 Å) at the Xpress beamline of Elettra Sincrotrone Trieste^43^. LaB_6_ was used for the calibration of the XRPD setup. The HP-XRPD patterns were collected using a membrane-driven symmetric Diamond Anvil Cell (DAC) (BETSA, France) with a culet size of 400 μm. A 200 μm thick stainless-steel sheet was indented to ~ 50 μm and drilled at the center with a 160 μm hole for sample loading. A 4:1 methanol-ethanol (ME) mixture was used as a pressure-transmitting medium (PTM). The ruby fluorescence in situ method monitored the pressure inside the DAC. A monochromatic circular beam of cross-sectional diameter 50 μm was used for HP-XRPD data collection. Dioptas software was utilized to convert powder-diffraction rings into 2θ intensity plots, and Rietveld refinement with GSAS-II software was used to calculate the structural parameters from the measured XRD patterns.

Ambient and HP Raman scattering measurements were conducted using a 532 nm (green) diode laser with 2400 lines/mm gratings in a customized Renishaw Raman confocal microscope at the Xpress beamline. The same DAC pressure cell used for HP-XRPD measurements was employed for HP Raman measurements, as discussed in the previous section. Detailed measurement procedures can be found in reference^27^. Because of the notch filter limitation, spectra were collected only above 110 cm^− 1^, and modes below this range were not accessible.

Ambient and high-pressure electrical resistivity ρ(T) was measured using a DC four-probe configuration in a cryogen-free closed-cycle refrigerator (CCR-VTI). For each temperature and pressure point, the polarity of the applied DC current was reversed, and the resistivity was calculated from the average of the measured voltages for positive and negative current directions. This procedure minimizes thermoelectric offsets and contact-related spurious voltages. Ambient pressure measurements were performed using a standard four-probe technique, with four electrical contacts made on the top of a rectangular sample (dimensions: 1.5 × 0.5 × 0.5 mm) using high-quality silver paste (Dupont: 4929 N) and copper wires (0.03 mm ϕ). Pressure-dependent electrical resistivity measurements were carried out using a Diacell CryoDAC-PPMS (Almax easyLab) pressure cell with 600 μm diamond anvils. The gasket was made of CuBe (Be 2%) with an initial thickness of 300 μm, indented to 100 μm. A micro electrical discharge machine (EDM) created a 300 μm hole at the center of the indentation. The insulation layer was prepared using a stycast 1266 and Al_2_O_3_ mixture with a thickness of ~ 10 μm, and a 200 μm hole was drilled at the center of the sample chamber. The four-probe *van der Pauw* method was employed to measure resistivity under high pressure. Electrical contacts on the ~ 90 μm x ~ 70 μm sample were made using gold wire (10 μm ϕ) with silver epoxy (Dupont: 6838), cured at 180 °C for 2 h in an argon gas atmosphere. Platinum foil (20 μm thickness) was used to connect the gold wire (10 μm ϕ) to external copper wires (0.03 mm ϕ). The copper wires were connected using pure silver paste (Dupont: 4929 N) and cured at room temperature for 16 h. The copper wires were soldered to a breadboard, and external shielded wires connected the breadboard to the constant current source and nano-voltmeter. Glycerine was used as a pressure-transmitting medium for better hydrostaticity. The pressure inside the cell was accurately measured using the ruby fluorescence technique (Optiprexx RubyLux, Almax easyLab). Three ruby chips were placed on the top diamond anvil, surrounding the sample when closing the diamond anvils. High Pressure Manager software (Almax easyLab) was used to monitor the pressure. The residual resistivity ratio (RRR) was calculated as RRR = ρ(300 K)/ρ(10 K), using the corresponding averaged resistivity values.

### Computational methods

The cage-like superconducting compound Y_5_Rh_6_Sn_18_ was investigated from first principles to analyze the pressure-induced changes in its electronic properties, resulting into the experimentally observed variation in the superconducting critical temperature. We have carried out an electronic structure calculation by using a planewave basis set with norm-conserving projector augmented wave (PAW) pseudopotentials as implemented in the Vienna ab initio simulation package (VASP)^44,45^. We have used the Perdew-Burke-Ernzerhof (PBE) exchange–correlation functional within the generalized gradient approximation (GGA)^46^ to represent the electronic correlations, together with the projector augmented-wave (PAW) method^47^. The plane-wave cut off energy is set as 500 eV. The valence electron configurations for the Y, Rh and Sn pseudopotentials are 4s^2^ 4p^6^ 4d^2^ 5s^1^, 4d^8^ 5s^1^ and 5s^2^ 5p^2^ respectively. Both the lattice parameters and the ionic positions were relaxed to determine the ground state configuration until the value of the Hellmann Feynman force on each ion is less than 10^− 4^ eV/Å. For all self-consistent electronic calculations, the total energy convergence criteria are kept as 10^− 5^ eV. A Monkhorst-Pack grid of 7 × 7 × 3 is used for Brillouin zone sampling to extract the density of states (DOS). We carried out non-spin-polarized calculations to obtain the ground state of the compound at different pressures by systematically reducing the unit cell volume while keeping the unit cell shape (symmetry) unchanged.

## Supplementary Information

Below is the link to the electronic supplementary material.

Supplementary Material 1

## Data Availability

All data generated or analysed during this study are included in this published article and its supplementary information files.
