# Supplementary material for: Impact of pressure on the structural, Raman, superconducting, and normal state resistivity properties of Y5Rh6Sn18 quasi-skutterudite single crystal
Source: Sci Rep. 2026 Mar 10;16:12933. doi: 10.1038/s41598-026-40887-8 (PMC13096136; doi:10.1038/s41598-026-40887-8)
Supplement: Supplementary file 1 — Supplementary Material 1 [file 41598_2026_40887_MOESM1_ESM.docx]

**Supplementary section**

Figure S1: Temperature dependence of the normalized electrical resistivity (ρ(T) / ρ(300 K)) of Sc_5_Rh_6_Sn_18_ (black open squares), Y_5_Rh_6_Sn_18_ at ambient pressure (red open circles), and Y_5_Rh_6_Sn_18_ at 10.6 GPa (blue open triangles). The inset shows a zoom over low temperature part, permitting clearly the visualization of the superconducting transition. Calculated RRR value for Sc_5_Rh_6_Sn_18_ and Y_5_Rh_6_Sn_18_ at ambient pressure are respectively 1.12 and 0.77.The RRR value for Y_5_Rh_6_Sn_18_ at 10.6 GPa is 0.92.

Figure S2: Measured powder x-ray-diffraction patterns of Sc_5_Rh_6_Sn_18_ sample under various pressures up to 11.7 GPa at room temperature.

Figure S3: (a) represent the Sc_5_Rh_6_Sn_18_ lattice parameters as a function of pressure. (b) depict unit cell volume as a function of pressure. The red solid line represents the second-order Birch-Murnaghan equation of state fitting.

Figure S4. Pressure dependence of the c/a ratio for Sc_5_Rh_6_Sn_18_ compound

Figure S5. Pressure dependence of the c/a ratio for Y_5_Rh_6_Sn_18_ compound
